# Robotic surgery in emergency setting: 2021 WSES position paper

**DOI:** 10.1186/s13017-022-00410-6

**Published:** 2022-01-20

**Authors:** Nicola de’Angelis, Jim Khan, Francesco Marchegiani, Giorgio Bianchi, Filippo Aisoni, Daniele Alberti, Luca Ansaloni, Walter Biffl, Osvaldo Chiara, Graziano Ceccarelli, Federico Coccolini, Enrico Cicuttin, Mathieu D’Hondt, Salomone Di Saverio, Michele Diana, Belinda De Simone, Eloy Espin-Basany, Stefan Fichtner-Feigl, Jeffry Kashuk, Ewout Kouwenhoven, Ari Leppaniemi, Nassiba Beghdadi, Riccardo Memeo, Marco Milone, Ernest Moore, Andrew Peitzmann, Patrick Pessaux, Manos Pikoulis, Michele Pisano, Frederic Ris, Massimo Sartelli, Giuseppe Spinoglio, Michael Sugrue, Edward Tan, Paschalis Gavriilidis, Dieter Weber, Yoram Kluger, Fausto Catena

**Affiliations:** 1 Unit of Digestive, Hepatobiliary, and Pancreatic Surgery, CARE Department, Henri Mondor University Hospital (AP-HP), Créteil, France; 2 Faculty of Medicine, University of Paris Est, UPEC, Créteil, France; 3 Department of Colorectal Surgery, Queen Alexandra Hospital, University of Portsmouth, Southwick Hill Road, Cosham, Portsmouth, UK; 4 First Surgical Clinic, Department of Surgical Oncological and Gastroenterological Sciences, University of Padua, Padua, Italy; 5 Department of Pediatric Surgery, Spedali Civili Children’s Hospital of Brescia, Brescia, BS Italy; 6 General Surgery, San Matteo University Hospital, Pavia, Italy; 7 Division of Trauma and Acute Care Surgery, Scripps Memorial Hospital La Jolla, La Jolla, CA USA; 8 General Surgery and Trauma Team, ASST Niguarda Milano, University of Milano, Milan, Italy; 9 General Surgery, San Giovanni Battista Hospital, USL Umbria 2, Foligno, Italy; 10 General, Emergency and Trauma Department, Pisa University Hospital, Pisa, Italy; 11 Department of Digestive and Hepatobiliary/Pancreatic Surgery, Groeninge Hospital, Kortrijk, Belgium; 12 Department of Surgery, Cambridge University Hospital, NHS Foundation Trust, Cambridge, UK; 13 Digestive and Endocrine Surgery, Nouvel Hôpital Civil, University of Strasbourg, Strasbourg, France; 14 IRCAD, Research Institute Against Digestive Cancer, Strasbourg, France; 15 Department of General and Metabolic Surgery, Poissy and Saint-Germain-en-Laye Hospitals, Poissy, France; 16 Department of General Surgery, Hospital Valle de Hebron, Universitat Autonoma de Barcelona, Barcelona, Spain; 17 Department of General and Visceral Surgery, Medical Center University of Freiburg, Freiburg, Germany; 18 Department of Surgery, Tel Aviv University, Sackler School of Medicine, Tel Aviv, Israel; 19 Department of Surgery, Hospital Group Twente ZGT, Almelo, Netherlands; 20 Department of Gastrointestinal Surgery, University of Helsinki and Helsinki University Hospital, Helsinki, Finland; 21 Unit of Hepato-Pancreato-Biliary Surgery, General Regional Hospital “F. Miulli”, Acquaviva delle Fonti, Bari, Italy; 22 Department of Clinical Medicine and Surgery, “Federico II” University of Naples, Naples, Italy; 23 Ernest E Moore Shock Trauma Center at Denver Health, University of Colorado, Denver, CO USA; 24 University of Pittsburgh School of Medicine, Pittsburgh, PA USA; 25 Visceral and Digestive Surgery, Nouvel Hôpital Civil, University of Strasbourg, Strasbourg, France; 26 Institute for Image-Guided Surgery, IHU Strasbourg, Strasbourg, France; 27 Institute of Viral and Liver Disease, INSERM U1110, Strasbourg, France; 28 3Rd Department of Surgery, Attikon General Hospital, National and Kapodistrian University of Athens (NKUA), Athens, Greece; 29 1St General Surgery Unit, Department of Emergency, ASST Papa Giovanni Hospital Bergamo, Bergamo, Italy; 30 Division of Digestive Surgery, University Hospitals of Geneva and Medical School, Geneva, Switzerland; 31 Department of Surgery, Macerata Hospital, Macerata, Italy; 32 IRCAD Faculty Member Robotic and Colorectal Surgery-IRCAD, Strasbourg, France; 33 Department of Surgery, Letterkenny University Hospital, Donegal, Ireland; 34 Department of Surgery, Trauma Surgery, Radboud University Medical Center, Nijmegen, Netherlands; 35 Department of HBP Surgery, University Hospitals Coventry and Warwickshire NHS Trust, Clifford Bridge Road, Coventry, CV2 2DX UK; 36 Department of Trauma Surgery, Royal Perth Hospital, Perth, Australia; 37 Department of General Surgery, Rambam Healthcare Campus, Haifa, Israel; 38 Department of General and Emergency Surgery, Bufalini Hospital-Level 1 Trauma Center, Cesena, Italy

**Keywords:** Emergency surgery, Robotic surgery, General surgery, Minimally invasive surgery

## Abstract

**Background:**

Robotics represents the most technologically advanced approach in minimally invasive surgery (MIS). Its application in general surgery has increased progressively, with some early experience reported in emergency settings. The present position paper, supported by the World Society of Emergency Surgery (WSES), aims to provide a systematic review of the literature to develop consensus statements about the potential use of robotics in emergency general surgery.

**Methods:**

This position paper was conducted according to the WSES methodology. A steering committee was constituted to draft the position paper according to the literature review. An international expert panel then critically revised the manuscript. Each statement was voted through a web survey to reach a consensus.

**Results:**

Ten studies (3 case reports, 3 case series, and 4 retrospective comparative cohort studies) have been published regarding the applications of robotics for emergency general surgery procedures. Due to the paucity and overall low quality of evidence, 6 statements are proposed as expert opinions. In general, the experts claim for a strict patient selection while approaching emergent general surgery procedures with robotics, eventually considering it for hemodynamically stable patients only. An emergency setting should not be seen as an absolute contraindication for robotic surgery if an adequate training of the operating surgical team is available. In such conditions, robotic surgery can be considered safe, feasible, and associated with surgical outcomes related to an MIS approach. However, there are some concerns regarding the adoption of robotic surgery for emergency surgeries associated with the following: (i) the availability and accessibility of the robotic platform for emergency units and during night shifts, (ii) expected longer operative times, and (iii) increased costs. Further research is necessary to investigate the role of robotic surgery in emergency settings and to explore the possibility of performing telementoring and telesurgery, which are particularly valuable in emergency situations.

**Conclusions:**

Many hospitals are currently equipped with a robotic surgical platform which needs to be implemented efficiently. The role of robotic surgery for emergency procedures remains under investigation. However, its use is expanding with a careful assessment of costs and timeliness of operations. The proposed statements should be seen as a preliminary guide for the surgical community stressing the need for reevaluation and update processes as evidence expands in the relevant literature.

## Background

Robotics represents the most technologically advanced approach in minimally invasive surgery (MIS). Its application has progressively gained acceptance in several surgical fields, being routinely used for elective urology, gynecology, digestive, and hepato-bilio-pancreatic surgery [1–8]. Conversely, robotic surgery in the emergency setting has not been explored, although some early experience has been reported in the literature [9–12]. Consequently, the issue regarding the role and potential applications of robotics for emergency procedures remains open. However, it deserves to be continuously monitored and updated in the future as evidence would emerge.

### Project rationale and design

The present position paper is supported by the World Society of Emergency Surgery (WSES) and aims to provide a systematic review of the literature investigating the use of robotics in emergency general surgery to develop consensus statements based on the currently available evidence and practice. The present document should be seen as a preliminary guide for the surgical community stressing the need for reevaluation and update processes as evidence expands in the relevant literature.

For the purpose of this WSES position paper, the organizing committee (composed of Fausto Catena, Nicola de’Angelis, and Jim Khan) constituted a steering committee (made up of 16 experts), who had the task of drafting the present position paper, and an international expert panel composed of 21 experts who were asked to critically revise the manuscript and position statements. The position paper was conducted according to the WSES methodology [13]. We shall present the systematic review of the literature and provide the derived statements upon which a consensus was reached, specifying the quality of the supporting evidence and suggesting future research directions.

## Systematic review

### Methods

#### Review question, selection criteria, and search strategy

The systematic review of the literature was performed following the Cochrane Collaboration specific protocol [14] and was reported according to the Preferred Reporting Items for Systematic Reviews and Meta-Analyses (PRISMA) statement [15].

The focus question was the following: *what are the applications and outcomes of robotics for general surgery in emergency settings?*

Studies reporting the use of a robotic surgical platform to manage general surgery emergencies and urgencies were searched in the following databases on June 30, 2021: MEDLINE (through PubMed), Embase, and the Cochrane Library. A specific research query was formulated for each database, using the following keywords and MeSH terms: emergency, emergency surgery, emergency setting, urgent, robotic surgery, robotic, robotics, robot-assisted, minimally invasive surgery, and minimally invasive surgery procedures.

According to the PICOS format, the following items were used as selection criteria for articles emerging from the literature search:

- P, population: adult patients requiring surgery in emergent/urgent settings.
- I, intervention: robotic or robot-assisted general surgery intervention.
- C, comparisons: laparoscopy or open surgery or no comparison.
- O, outcome(s): operative and postoperative surgical outcomes.
- S, study design: due to the expected paucity of studies on the topic, all types of comparative study, but also case series and case reports were considered aiming to provide the most exhaustive picture of the current evidence and practice in robotic emergency general surgery.

The research was limited to studies published in English.

The literature search and selection were performed by two independent reviewers (GB and FM), who also screened the reference list of the selected articles to potentially include additional studies. First, all records from merged searches were reviewed for relevance concerning title and abstract. Records were removed when both reviewers excluded them. Otherwise, the disagreement was resolved via discussion or with the intervention of a tiebreaker (NdeA). Both reviewers then performed an independent full-text analysis, which allowed to finally include or exclude the preselected article.

#### Data extraction and synthesis

Data extraction was performed by filling in an electronic spreadsheet, which included the following items: first author’s name, year of publication, scientific journal, type of study, number of patients, pathological state requiring surgical intervention, type of surgical intervention, surgical approach, operative surgical outcomes, and postoperative surgical outcomes. The risk of bias in the selected studies was assessed by using validated systems according to the type of study design [16–18].

### Results

#### Literature search and selection

**Fig. 1:**
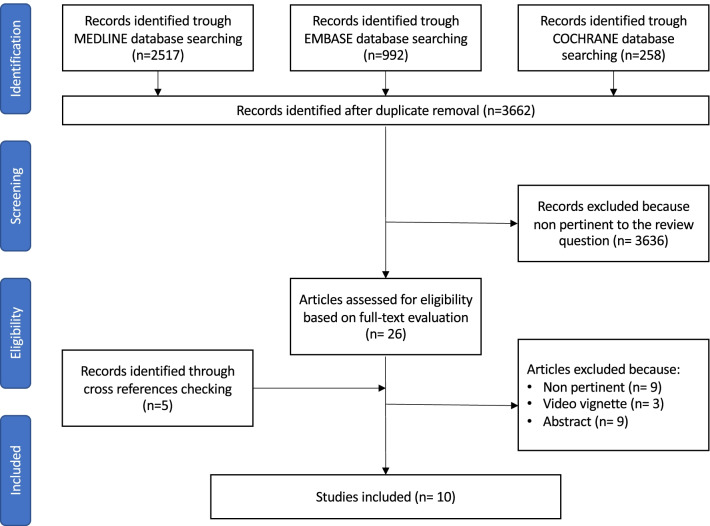
Flowchart of the literature search and selection

The initial search yielded 3767 results; after removing duplicates, 3662 articles were screened for eligibility based on title and abstract, and 31 articles were retrieved for a full-text evaluation. A total of 10 studies fulfilled the selection criteria and were finally included in the review (Fig. 1).

#### Study characteristics

The selected 10 studies were published between 2012 and 2021. They consisted of 5 cohort studies and 5 case reports conducted in Europe (*n* = 3) and North America (*n* = 7). The characteristics of the examined studies are summarized in Table 1. Overall, they considered 279 patients.

**Table 1:** Studies reporting on urgent/emergent general surgery interventions performed with a robotic approach

References	Study design	Nb. of patients	Time period	Pathology	Intervention	Robotic platform	Outcomes	Results
**Comparative studies**								
Hosein et al. [21]	Retrospective cohort study	131 robotic cases 719 open cases 1517 laparoscopic cases	January 2015–December 2017	Hiatal hernia	Hiatal hernia repair	Unknown	Perioperative outcomes	Trend towards better outcomes in minimally invasive surgery (laparoscopic and robotic) as compared to open approach
Kubat et al. [22]	Retrospective cohort study	76 robotic (elective) cases 74 robotic (urgent) cases	May 2001–August 2013	Acute cholecystitis, choledocholithiasis, severe chronic cholecystitis	Robotic single-site cholecystectomy	Intuitive Surgical da Vinci Si™	Perioperative outcomes and learning curve	Robotic single-site cholecystectomy is safe and can be applied to urgent and elective settings with acceptable perioperative outcomes. Learning curve of 50 cases
Robinson et al. [12]	Retrospective cohort study	4 robotic cases 0 laparoscopic cases	2015–2019	Perforated gastrojejunal ulcers following Roux-en-Y gastric bypass surgery	Repairs of perforated gastrojejunal ulcers	Intuitive Surgical da Vinci Si™/Xi™	Perioperative outcomes	Outcomes are non-inferior to laparoscopy with the use of the robot. In-room-to-surgery time is reduced in the robotic group. Costs are greater in the robotic group
Anderson et al. [9]	Retrospective cohort study on a prospective database	6 robotic cases 13 laparoscopic cases	February 2015–February 2017	Severe acute ulcerative colitis	Subtotal colectomy	Intuitive Surgical da Vinci Xi™	Feasibility and perioperative outcomes	Robotic subtotal colectomy has similar perioperative outcomes of laparoscopic subtotal colectomy
**Case series and case reports**								
Kudsi et al. [25]	Case series	34 robotic cases	February 2013–November 2019	Ventral hernia	Robotic ventral hernia repair	Intuitive Surgical da Vinci™	Perioperative outcomes and mid-term follow-up	Robotic ventral hernia repair is effective in the emergency setting
Ceccarelli et al. [21]	Case series	3 robotic cases	December 2009–December 2019	Giant hiatal hernia	Hernia repair ± fundoplication	Intuitive Surgical da Vinci Xi™	Perioperative outcomes	Uneventful postoperative course in patients, one patient developed an antrum stenosis due to preoperative mucosal ischemia
Milone et al. [23]	Case series	3 robotic cases	2019	Moderate acute calculous cholecystitis	Robotic cholecystectomy	Intuitive Surgical da Vinci Si™	Perioperative outcomes	Uneventful postoperative course
Sudan et al. [24]	Case report	Robotic cases	2001	Complications of biliopancreatic diversion with duodenal switch	Robotic strictureplasty Robotic suture of duodenal stump	Intuitive Surgical da Vinci Si™	Perioperative outcomes	Uneventful postoperative course
Felli et al. [10]	Case report	1 robotic case	2014	Hemorrhagic right colon cancer	Robotic right colectomy with double-barreled ileocolostomy	Intuitive Surgical da Vinci Si™	Perioperative outcomes	Uneventful postoperative course. At 5 months of follow-up, no recurrence was noted
Pedraza et al. [19]	Case report	1 robotic case	2001	Iatrogenic colonoscopy perforation	Robotic colorrhaphy	Intuitive Surgical da Vinci Si™	Perioperative outcomes	Uneventful postoperative course

Three studies reported interventions of colorectal surgery [9, 10, 19], two studies reported on hiatal hernia surgery [20, 21], two studies reported on gallbladder surgery [22, 23], two studies reported on bariatric surgery [12, 24], and one study reported on abdominal wall surgery [25]. Only one case was a cancer-related emergency [10].

#### Qualitative synthesis of the literature

1. Robotics in emergency colorectal surgery

An early preliminary report of an emergent robotic repair of a colonic iatrogenic perforation was published by Pedraza et al. in 2012 [19]. The authors showed that such a procedure was feasible and successful. In 2014, Felli et al. [10] described the case of an 86-year old woman who underwent a robotic right colectomy for a bleeding ascending colon neoplasia. The surgery was uneventful and the reported postoperative outcomes were excellent. More recently, Anderson et al. [9] published a matched case–control study focusing on the use of robotics for urgent subtotal colectomies in patients presenting with ulcerative colitis. The results showed similar short-term outcomes for robotic and laparoscopic approaches.

2. Robotics in emergency hiatal hernia surgery

Over the last years, two groups published their early experience with robotic surgery for emergency hiatal hernia repair. In a case series of 3 patients undergoing robotic surgery for complicated giant hiatal hernia, Ceccarelli et al. [21] showed that postoperative outcomes were good. The authors suggested that the potential advantages of robotics over a conventional laparoscopic approach were mainly related to the surgeon’s comfort and precision during the intervention. Hosein et al. [20] performed a cohort-based analysis using data from the 2015–2017 Vizient clinical database, which included inpatient data from over 300 hospitals in the USA. Trend analysis demonstrated that laparoscopy was the most common approach in emergency hiatal hernia repair, representing 64.09% of cases, followed by the open (30.38%) and the robotic approach (5.53%). Concerning operative and postoperative outcomes, a trend was also observed for better outcomes in case of MIS (laparoscopy or robotic) hiatal hernia repair as compared to open surgery.

3. Robotics in emergency gallbladder surgery

In 2016, Kubat et al. [22] published a retrospective case series of 76 elective and 74 urgent robotic single-site cholecystectomies. The authors reported good perioperative outcomes, concluding that this approach was safe and efficient. In 2019, Milone et al. [23] described a case series of 3 patients who underwent robotic cholecystectomy for acute cholecystitis. The reported perioperative outcomes were excellent and the authors recommended the introduction of robotics in emergency settings in order to validate their preliminary results.

4. Robotics in emergency bariatric surgery

The first report of robotic emergency surgery after complicated robotic biliopancreatic diversion with duodenal switch was published by Sudan et al. in 2012 [24]. The robotic approach was preferred over open surgery in the management of postoperative complications in order to preserve the benefits of the previous MIS approach. The authors highlighted how the adoption of the robotic platform was useful in a patient in order to identify the damage and to repair it. More recently, Robinson et al. [12] published a retrospective cohort study comparing emergent laparoscopic and robotic gastrojejunal ulcer repair. The authors showed that in-room-to-surgery-start time was significantly reduced in the robotic group. Additionally, perioperative outcomes were in favor of the robotic approach, although not significantly different. However, robotic surgery was significantly more expensive than laparoscopy.

5. Robotics in emergency abdominal wall surgery

In 2020, Kudsi et al. [25] published an article on the perioperative and mid-term outcomes of 34 patients who underwent emergency robotic ventral hernia repair with different techniques between 2013 and 2019. With a 20.5% rate of minor postoperative complications (Clavien-Dindo grades I-II), a 11.7% rate of major postoperative complications (Clavien-Dindo grades III-IV), and only one (2.9%) patient experiencing hernia recurrence, the authors concluded that robotic ventral hernia repair was associated with promising results and overall feasibility in emergency settings, to be tested in further long-term follow-up studies.

#### Evaluation of the quality of evidence

Five out of 10 selected studies were retrospective cohort studies and were evaluated according to the NOS [18]. Two studies received a score of 8/9 [9, 12], one study was graded 7/9 [20], and two studies had a score of 6/9 [22, 25] (Table 2). The remaining studies were evaluated according to the tool described by Murad et al. [16]. All studies received a score of 6/8 [10, 19, 21, 23, 24] (Table 3).

**Table 2:** Quality assessment for the selected retrospective cohort studies according to the Newcastle Ottawa Scale (NOS)

References	Selection	Comparability	Outcome/exposure	Overall score
Kubat et al. [22]	***	–	***	6/9
Anderson et al. [9]	****	*	***	8/9
Kudsi et al. [25]	***	–	***	6/9
Hosein et al. [21]	***	*	***	7/9
Robinson et al. [12]	****	*	***	8/9

**Table 3:** Quality assessment for the selected case series/case reports according to Murad et al. [16]

References	Selection	Ascertainment	Causality	Reporting	Overall score
Pedraza et al. [19]	*	**	**	*	6/8
Sudan et al. [24]	*	**	**	*	6/8
Felli et al. [10]	*	**	**	*	6/8
Milone et al. [23]	*	**	**	*	6/8
Ceccarelli et al. [21]	*	**	**	*	6/8

## Position statements

Following a comprehensive literature review and the summary of current scientific evidence on the applications of robotics for emergency general surgery procedures, the following position statements (PS) were put forward. For each statement, the supporting literature, the level of evidence, and the strength of the consensus are indicated. The level of evidence is classified according to the GRADE system (https://training.cochrane.org/introduction-grade). For each statement, the consensus was assessed through a web survey (by means of a Google Form) open to all members of the steering committee and panel of experts and to the members of the Board of Governors of the WSES. If a statement reached < 70% of agreement, it was rediscussed via email or videoconference, modified, and resubmitted to the experts’ vote until a consensus was reached.

The experts involved were also asked to describe their current practice. The great majority (82.6%) worked in a hospital equipped with a robotic surgical platform. However, the access to the robotic surgical system for emergency procedures appeared to be limited, with difficult availability (39.1%) only during the day (13%), or not available at all (43.5%).

***PS-1. Robotic surgery in emergency settings is highly dependent on the surgeon’s experience and should only be performed in an appropriately equipped operating room with trained nursing staff.***

*Supporting literature*

Robotic surgery requires a high level of technical expertise when compared to open or even laparoscopic surgery. A complete specialized training is required to be proficient in performing standardized surgical interventions associated with acceptable operative and postoperative outcomes [26]. In a recent article, Thomas et al. [27] analyzed the robotic colorectal surgery activity of a tertiary colorectal unit and concluded that success relies on a structured training curriculum, a dedicated surgical team, the institution’s support, and many other variables in addition to the training at the robotic console itself. The adoption of the robot in the emergency setting does not change the rules of the game. Rather, it enhances the need for a safe and efficient strategy starting from the standardization of the robotic platform setting and docking, up to the execution of the surgical procedure. In order to successfully perform emergency cases with a robotic system, the on-call surgical team must be adequately trained with robotic technology. As reported by Robinson et al. [12] in a case series of 24 robotic emergency bariatric surgeries, which were compared to 20 laparoscopic procedures, the surgeon who adopted the robotic approach was the same in all cases. It is the proof that a specific attitude of the operator is fundamental. However, it also highlights the need for a “can do” attitude from the entire surgical team [28]. The importance of the shared viewpoint is reinforced by Sudan et al. [24] who described the adoption of the robotic platform during the night and during the weekend in order for the staff to be comfortable with this technology. In addition, proper team work and communication in such a challenging workspace are required [29] as much as the completion of the learning curve for the entire surgical team [30]. The ideal operating room team in an emergency setting should be made up of the first operating surgeon with an extensive expertise in robotic surgery, an assisting surgeon familiar with the robotic technology, and a scrub nurse dedicated to the robotic program. All team members should work in a simulation environment before starting a robotic emergency surgery program.

Limitations linked to the adoption of robotic surgery in emergency settings are related to the time required for robotic setting and docking and the accessibility of the robotic platform for emergency surgical units. Concerning the time issue, Robinson et al. [12] reported that, when the entire team is appropriately trained and prepared, the in-room-to-surgery-start time is reduced and has no significant impact on the overall duration of the scheduled emergency procedure. However, in this study, the authors highlighted how the majority of the staff were familiar with the robotic technology, and there were no limitations to its accessibility. This may not be the case for all emergency care units, and trained nursing staff may not be always available during night shifts. A good coordination between the hospital administration, the surgeons, and the staff is the key point to have an efficient and extensive organization for the use of robotic technology, also in emergency surgery scenarios.

- *Level of evidence:* case reports and case series → expert opinion
- *Strength of consensus (based on the survey evaluation): 100%*

***PS-2. Robotic surgery in emergency settings may be considered in highly selected clinically stable patients only.***

*Supporting literature*

Due to the very limited evidence in the literature and the consensus that robotic surgery required a high level of expertise for the operating surgeon and the entire surgical team, particularly if performed in emergency settings, it should be considered for clinically stable patients only.

A recent review [31] on the anesthetic aspects of robotic surgery suggested that when the surgical team gains confidence, even more complex operations or patients with comorbidities can be considered candidates for the robotic approach. A precise preoperative assessment based on a case-by-case evaluation, and multidisciplinary decision-making are crucial to guarantee the choice of the most indicated surgical strategy. Even if a comprehensive preoperative assessment is not always possible in emergency situations, a careful patient selection is advised in order not to expose frail or unstable patients to longer emergency procedures or unnecessary complications related to the surgical technique.

Indeed, in unstable patients or patients with cardiopulmonary comorbidities, the adoption of MIS with the need for carbon dioxide insufflation may result in a higher intra-abdominal pressure and hypercarbia with metabolic and respiratory changes which may be deleterious [32]. Osagiede et al. [11] showed that the presence of a metastatic disease and the higher number of comorbidities negatively influenced the adoption of MIS in emergency colorectal cancer surgery. Likewise, Arnold et al. [33] demonstrated that the adoption of MIS is confined to physiologically clinically stable patients while those with abdominal gross contamination or severe infectious processes are more prone to undergo open surgery. Despite this selection bias, when the results are corrected for preoperative risk factors, the adoption of laparoscopy is associated with a reduced wound infection rate, risk of death, and length of hospital stay.

Recently, emergency laparoscopy was evaluated as a valid approach to the treatment of perforated diverticulitis with generalized peritonitis [34], iatrogenic colonoscopy perforations [35], and perforated peptic ulcers [36]. In addition, in simple cases of adhesive small bowel obstruction, a laparoscopic approach may be beneficial despite the considerable risk of conversion to open surgery and the higher probability of bowel injuries [37]. In all of the abovementioned pathological states, the prerequisite for a safe minimally invasive treatment is the selection of a stable patient.

In terms of anesthetic management in emergency settings, the robotic approach can be considered as an alternative to laparoscopy because it does not change the risk exposure but it may be associated with longer operative times if the surgical team is not properly trained. Additional costs must also be considered. Further studies are necessary in order to clarify the future role of a low pressure pneumoperitoneum in emergency robotic surgery [38].

- *Level of evidence:* case reports and case series → expert opinion
- Strength of consensus: 94.6%

***PS-3. Robotic surgery may be considered in challenging situations, which are foreseen as a reason for conversion to open surgery if operating in laparoscopy.***

*Supporting literature*

The available literature suggests that the main potential advantages of robotic surgery over laparoscopy are related to suturing and dissection. In case of emergency robotic surgery, the published studies described the following procedural steps: hiatoplasties [20, 21], ventral suturing or mesh fixations [25], colonic suturing [19], duodenal stump suturing [24], strictureplasty [24], and dissection of inflamed gallbladder [22, 23] or colon [9]. All of these tasks are particularly challenging in laparoscopic surgery and they often lead to conversion to open surgery, which can also be a source of postoperative complications [39, 40]. The technological advances of the robotic surgical platform, such as deep magnification, 3D stereoscopic vision, a stable field with elimination of physiological tremors, motion scaling, and improved ergonomics as compared to laparoscopy, may contribute to facilitate the performance of some difficult procedural steps and reduce the risk of conversion. However, this remains to be proven, especially for surgical interventions performed in emergency settings.

- *Level of evidence:* case reports and case series → expert opinion.
- *Strength of consensus: 83.8% (based on the survey evaluation)*

***PS-4. In a near future, robotic surgery may offer the advantage of telementoring and telesurgery, which could be useful to promote a safe and standardized application of robotics, also in low-volume centers or specific environments.***

*Supporting literature*

One of the limitations of laparoscopic surgery is the absence of telementoring during a difficult procedure. Even if communicating systems dedicated to telementoring are available, no opportunity for the direct control of movements is present in laparoscopy. In robotic surgery, an in-person mentoring can be performed if a second robotic console is present in the hospital (such as telestration or tele-assisted surgery). In a near future, it can be expected to perform telementoring during elective and emergency robotic procedures. After the first transatlantic robot-assisted surgery performed by Jacques Marescaux in 2001 [41], the surgical community was waiting for a routine use of telesurgery which, however, was not feasible due to technical limitations. Today, thanks to the evolution of telecommunications, namely fifth generation (5G) networks, there is a growing opportunity for a surgeon with a proven expertise in the field to remotely operate on a distant patient [42, 43]. A digital connection with a reference center which can evaluate the case, suggest a solution, and eventually manage the surgical situation if need be, represents a powerful tool, especially in emergency settings. Indeed, in emergency surgery where a maximal experience improves outcomes, it would be beneficial to have a mentor observing and remotely participating in the intervention. Additionally, this technology could be applied to provide surgical care to rural areas, to establish surgical collaborations, and to eliminate the shortage of surgeons. This is also applicable for specific environments, such as in the space station, where an emergency medical condition has to be managed by a trained component of the crew, or close to a battlefield, where the surgeon may operate at a safe distance, or again at the bottom of the ocean [44]. Telesurgery could well be an option in such situations.

However, these applications conceal some limitations in terms of global network development, legal and ethical issues, costs, and cyber security. These issues are under examination. However, despite the current skepticism, it is unquestionable that robotic surgery can have a pivotal role in developing telemedicine and telesurgery [45, 46].

- *Level of evidence:* case reports and case series → expert opinion
- *Strength of consensus: 89.2% (based on the survey evaluation)*

***PS-5. The use of robotic surgery for unscheduled and urgent operations needs to be implanted without creating scheduling conflicts in the occupation of the operating room. Moreover, the increased costs need to be justified in the context of an efficient implementation of robotic surgery. Currently, the availability and accessibility of the robotic platforms for emergency care surgical units are very limited.***

*Supporting literature*

A consistently growing number of hospitals, mainly tertiary care and university-based hospitals, are acquiring a robotic surgical platform in order to satisfy daily requests and advertise the most advanced technology. The robotic platform is often shared between different specialties, subsequently limited in terms of availability for a single surgical field and not adaptable to changing schedules. In this perspective, several reports suggested that the use of the robotic surgical platform by experienced teams could be prolonged to night hours and even to the weekend. This approach was called “after hours” by Sudan et al. [24], whose report aimed to highlight the potential of a robotic system which is available 24 h/7 days per week. The availability of the platform during the night shift could potentially favor a more cost-effective use of the robotic system. However, this remains very limited and, as previously highlighted, a proper attitude and excellent training of the entire team are key to guarantee surgical proficiency and efficiently implement robotic surgery for emergency procedures.

Concerns for the adoption of robotics for emergency surgeries also persist in relation to the increased costs that a robotic surgical procedure implies also need to be justified in the context of an efficient implementation of robotic surgery.

- *Level of evidence:* case reports and case series → expert opinion
- *Strength of consensus: 89.2% (based on the survey evaluation)*

***PS-6. The development of new modular robotic platforms may contribute to increase the applications of robotic surgery in emergency settings.***

*Supporting literature*

The surgical marketplace was recently enhanced with several different robotic platforms either approved for human use, such as the CMR Versius (Cambridge Medical Robotics, Cambridge, UK) and the Distalmotion Dexter (Distalmotion, Epalinges, Switzerland) or under approval, such as the Medtronic Hugo (Medtronic Inc., Minneapolis, USA). Most of them share the opportunity of switching from a conventional laparoscopic setting to a robot-assisted one. This key point, which could be less relevant in elective surgery, should be carefully considered when approaching emergency surgery. In fact, when no specific port placement is required, the surgeon can simply use a different approach depending on the procedural step and on his/her own ability. In addition, these robotic platforms offer an improved vision with advanced near-infrared imaging, not routinely available in laparoscopic surgery. The objective evaluation of tissue anatomy or perfusion could limit the surgical bias in emergency settings by mitigating the personal opinion [47, 48].

In the future, advances in surgical technologies will offer multiple new opportunities, which are currently under development, like hyperspectral imaging [49] and robotic single-port surgery [50]. Their potential applications and outcomes in emergency surgery need to be evaluated and updated once evidence is available.

- *Level of evidence:* case reports and case series → expert opinion
- *Strength of consensus: 94.6% (based on the survey evaluation)*

## Research agenda

The experts recognized that there is a substantial lack of evidence to support the use of robotic surgery for emerging general surgery procedures. For this reason, a research agenda has been proposed.

- Observational (cohort study, case–control) and interventional studies are anticipated to investigate the applications and outcomes of robotic surgery in emergency settings and to compare them with those obtained with laparoscopy and open surgery.
- Future studies should evaluate patient preferences considering patient-related outcome measures (PROMs), including pain evaluation and mid-/long-term quality of life.
- Future studies should evaluate the cost-effectiveness of robotic surgery implementation in emergency settings at hospital level (e.g., scheduling conflict alleviation) and at the level of the healthcare system (e.g., length of hospital stay, productivity losses, reimbursement systems).
- Future studies should evaluate the applicability of the robotic surgical platforms to perform telementoring and telesurgery, which are theoretically promising technologies to expand the applications of robotic surgery.

With the aim to enrich the available evidence and fill knowledge gaps, the WSES plans to launch an open registry on emergency robotic general surgery. The WSES calls for an international participation, which is essential to gather sufficient data and obtain generalizable results.

The establishment of a dedicated registry is also mandatory to perform a deep analysis on this technique, in order to define the following: characteristics of the patient candidate for emergency robotic procedures, operative and postoperative outcomes, PROMs, minimum requisites in terms of personnel and equipment, cost-effectiveness, and ethical issues.

## Discussion

Hospitals that are currently equipped with a robotic surgical platform need to implement it efficiently. The role of robotic surgery for emergency procedures remains under investigation. However, its use is expanding despite the lack of evidence-based guidelines. In this scenario, the WSES wished to provide this position paper to the surgical community. This position paper summarizes the current evidence and practice and proposes consensus statements to be reevaluated and updated as the evidence in the supporting literature emerges. For now, the experts recommend a strict patient selection while approaching emergent general surgery procedures with robotics. However, an emergency setting should not be seen as a contraindication for robotic surgery if adequate training of the operating surgical team is available. When such prerequisites are met, robotic surgery can be considered safe and feasible, and surgical outcomes related to an MIS approach are expected. Finally, the application of the robotic surgical platform may grow with improvements in telementoring and telesurgery, which are particularly valuable in emergency settings.

## Data Availability

There are no data from individual authors that reach the criteria for availability.

## Abbreviations

MIS: Minimally invasive surgery
NOS: Newcastle–Ottawa Scale
PROM: Patient-related outcome measures
PS: Position statements
WSES: World Society of Emergency Surgery
